# Grading of Hirsutism in Females: A Pilot Study

**DOI:** 10.7759/cureus.110863

**Published:** 2026-06-15

**Authors:** Resmi C.R., Muralee Damodaran, Usha V Menon, K.T. Moly, Sunil M, Nishath Hamza

**Affiliations:** 1 Department of Health Science Research, Amrita Institute of Medical Sciences, Amrita Vishwa Vidyapeetham, Kochi, IND; 2 Department of Endocrinology, Amrita Institute of Medical Sciences, Amrita Vishwa Vidyapeetham, Kochi, IND; 3 Department of Nursing, Amrita Institute of Medical Sciences, Amrita Vishwa Vidyapeetham, Kochi, IND; 4 Department of Clinical Genomics, The Royal Marsden Hospital NHS Foundation Trust, Surrey, GBR

**Keywords:** endocrinology, hirsutism, hyperandrogenism, modified ferriman-gallwey score, polycystic ovary syndrome

## Abstract

Background: Hirsutism is a common clinical manifestation of hyperandrogenism in women of reproductive age and may indicate underlying endocrine disorders such as polycystic ovary syndrome and thyroid dysfunction. Despite its clinical and psychosocial implications, data on its prevalence and severity in young, non-clinical populations remain limited.

Objective: To assess and grade the severity of hirsutism among young females aged 18-24 years using the modified Ferriman-Gallwey (mFG) scoring system.

Methodology: A pilot quantitative cross-sectional study was conducted among 25 young females using convenience sampling in a selected college in Kerala, India. Data on sociodemographic characteristics, menstrual history, comorbidities, and cosmetic practices were collected using a semi-structured proforma. Hirsutism severity was evaluated using the mFG scoring scale. Participants were provided with a standardised scoring sheet and asked to assess their body hair growth visually.

Result: The findings revealed that 48% of participants had normal scores, 32% had mild, and 20% had moderate-to-severe hirsutism. A considerable proportion reported menstrual irregularities and endocrine comorbidities, particularly polycystic ovary syndrome and thyroid disorders, consistent with existing literature.

Conclusion: Hirsutism is common among young females and may be associated with underlying endocrine dysfunction. Early screening using simple tools like the mFG score and multidisciplinary evaluation is essential, while larger studies are required to validate these findings.

## Introduction

Hirsutism, which is the excess terminal hair growth in secondary sexual parts of the body in females, occurs in about 5-10% of women of reproductive age and is an everyday clinical phenomenon of androgen excess or increased follicular sensitivity to existing androgens [1]. Clinical manifestation of the condition is highly heterogeneous, with mild cosmetic issues on one end and severe phenotypes characterised by systemic dysfunction of the endocrine system at the other end [2]. Hirsutism is often concomitant with previously mentioned physical symptoms, such as menstrual changes, metabolic disruptions, and body mass index changes, such as normal weight, overweight, and obesity [3]. Pathophysiology is mainly associated with hyperandrogenism, which can be caused by ovarian, adrenal, or idiopathic origins and thus a systematic clinical assessment is required to determine the aetiology of hyperandrogenism [4].

Polycystic ovary syndrome (PCOS) is the most common cause of hirsutism and accounts for the majority of cases, with studies reporting that hirsutism is present in approximately 65-75% of women with the condition, making it one of the most common clinical manifestations of hyperandrogenism in PCOS [5]. It is linked to metabolic abnormalities, such as insulin resistance and obesity, and tends to cause long-term reproductive and cardiovascular complications [6]. Less common causes of aetiology include non-classic congenital adrenal hyperplasia, Cushing syndrome, thyroid dysfunction, and hyperprolactinemia that necessitate differentiation as they have different management pathways [2].

The current literature shows that endocrinopathies are present in a significant proportion of women with hirsutism, with prevalence rates ranging from 60% to 95%, highlighting the importance of endocrine assessment in these patients [7]. Among them, PCOS is always the most common underlying cause, followed by idiopathic hirsutism, which has normal androgen levels but a high sensitivity in the periphery [8]. Epidemiological analyses also indicate that androgen-related disorders occur in approximately 10% of women in the world, and as a clinical indicator of more widespread endocrine derangement, hirsutism has a strong public health impact [9].

Several studies have highlighted the usefulness of the modified Ferriman-Gallwey (mFG) scoring system as a consistent and efficient method for assessing the severity of hirsutism in different populations [10]. However, ethnic variations in hair growth patterns should be considered, and the applicability of ethnicity-specific mFG cutoff scores for the Indian population requires further evaluation. Despite reported variations in cutoff values across different ethnic and population groups, the scoring system enables objective clinical assessment and facilitates comparison across studies [11]. The scoring system has made it possible to perform objective clinical evaluation and compare across studies, even though ethnicity and population variations in cut-off values have been recorded [11]. Also, the study shows that hirsutism has a high psychosocial burden, such as impaired self-esteem, anxiety, and poor quality of life, which supports the importance of identifying it early on and addressing it through overall management approaches [12]. The described treatment methods can be found in the literature that promotes a multimodal approach to treating the condition using lifestyle changes, pharmacological therapy, and cosmetic treatment [13]. Moderation and lifestyle adjustments have proven useful in lowering the androgen levels and clinical outcomes, especially in PCOS patients [14]. Pharmacological therapy with anti-androgens and hormonal therapy is the key to managing it, whereas cosmetic surgeries are used as adjuncts to the aesthetic side of the disorder [15,16].

Although the aetiology and management of hirsutism have been widely studied, there is still limited evidence regarding young adult females, particularly those in late adolescence and early reproductive age [17]. Focusing on college-aged females is important because hormonal and menstrual irregularities often emerge during this period, and concerns about appearance, self-esteem and quality of life can significantly affect their psychological and social well-being. Early identification in this age group may also help in timely screening and management of underlying endocrine disorders. The majority of the studies have focused on larger age population groups involved in reproductive age or clinically diagnosed cases, hence limiting the information on early-stage or subclinical presentations [18,19]. Besides, some inconsistencies in the prevalence and severity of hirsutism among various demographic and ethnic groups are not fully investigated, particularly in community-based contexts [20].

Evidence on hirsutism severity distribution with standardised measures like the mFG score in non-clinical groups is also limited. This lapse limits the capacity to establish initial trends, risk situations, and the possible development of the condition before seeking clinical care. Also, the psychosocial and lifestyle associates of hirsutism among younger age groups are inadequately examined, despite their possible impact on the perception of the disease and its management consequences.

Objectives of the study

The present study aims to assess and grade the severity of hirsutism among young females aged 18-24 years using the mFG scoring system, evaluate the association between mFG scores and selected clinical variables, and provide baseline data for future research and screening strategies.

## Materials and methods

Study design and setting

This pilot quantitative cross-sectional study aimed to test the viability of measuring hirsutism severity in a group of young adult females and to obtain preliminary data for use in future large-scale investigations. Although the mFG score is an established clinical scoring system, this pilot study was conducted to assess its feasibility and applicability for grading hirsutism in a young, non-clinical college-based population, where early or subclinical presentations may otherwise remain unrecognised. Being a pilot study, the main focus was to assess the applicability of the data collection tools, evaluate participants' reactions, and identify hirsutism distribution patterns in a specific population.

Study population and sampling technique

The study population comprised young women aged between 18 and 25 years attending the selected college. To find the participants, a non-probability convenience sampling method was used, which was appropriate for this pilot study. However, convenience sampling may introduce selection bias, as the selected participants may not fully represent the broader population. Individuals who choose to participate may differ from non-participants in characteristics such as awareness of symptoms, health concerns, or willingness to seek healthcare. As a result, the sample's representativeness may be limited, and the findings may not be fully generalizable to all young females. The study included 25 participants, a sample size considered feasible and suitable for assessing feasibility and conducting a preliminary analysis. The pilot nature of the study suits external validity, but offers the necessary information about the study design, the possibility of recruiting the required number of participants, and the variability of data, which are important in planning a large-scale, hypothesis-driven research.

Eligibility criteria

The inclusion criteria included females aged between 18 and 25 years who provided informed consent, regardless of marital status. An exclusion criterion was used to reduce the confounding variables that might influence the levels of androgens or hair development. Those who were not present during data collection, pregnant or lactating women, and those with severe congenital or chronic systemic illnesses (such as congenital endocrine disorders, chronic kidney disease, liver disease, or malignancy) were excluded from the study. However, participants with PCOS or thyroid disorders were included, as these conditions are closely associated with hirsutism. Also, individuals who were on long-term medication like corticosteroids or hormonal medications were not included. These criteria were stipulated to obtain a relatively homogeneous sample for assessing hirsutism patterns in this pilot study.

Data collection instruments

To conduct this pilot study, data were collected using a pre-designed semi-structured proforma designed to gather the relevant information. The tool consisted of 11 questions covering sociodemographic and clinical variables, including age, height, weight, BMI, age at menarche, menstrual irregularities, premenstrual symptoms, family history, comorbid conditions, medication use, and cosmetic practices. Clearance and consistency of response and administration were to be evaluated using the instrument. BMI was determined based on traditional anthropometric measurements, and it helped assess its clinical relationship with the degree of hirsutism. Eligible students who were available during the data collection period were approached; the purpose of the study was explained, and participation was requested only after confirming willingness and eligibility. Written informed consent was obtained before data collection.

Assessment of hirsutism

Hirsutism severity was assessed using the mFG scoring system, originally described by Ferriman and Gallwey [21]. Participants assessed terminal hair growth by self-scoring the nine androgen-sensitive body regions using the mFG scale with the help of standard reference images. The scoring method and 0-4 grading pattern were explained by the investigator before assessment to improve consistency in self-assessment. The regions assessed included the upper lip, chin, chest, upper back, lower back, upper abdomen, lower abdomen, upper arms, and thighs. Each area was scored from 0 to 4 based on the density and distribution of terminal hair growth, giving a total possible score of 0-36. Scores were categorised as <8 (normal), 8-15 (mild hirsutism), and >15 (moderate to severe hirsutism).

Data collection procedure

Data collection was done with institutional permission in advance. The subjects were recruited in the college environment, and the purposes of the study were communicated. Participation was informed by means of written consent. The semi-structured proforma was administered, and the respondents completed the questionnaire, including a self-evaluation using the mFG scale. It took an hour or two to complete every session. The pilot study design permitted an evaluation of the participants' level of involvement, the comprehensiveness of the inquiries, and the time taken to respond, which are important for improving the approach in future research.

Ethical considerations

The study adhered to ethical standards. The involvement was voluntary, and informed consent was taken from all the participants. Anonymity and confidentiality were guaranteed due to the absence of gathering any identifying data and safe data management. The participants were told that they had the right to withdraw at any time without penalty. Privacy was also observed in the process of data collection, as the topic was sensitive. Before the commencement of the study, the institutional authority approved the study protocol, and this fact guaranteed that it adhered to ethical principles of conducting research with human subjects.

## Results

Sociodemographic characteristics of the participants

The study included 25 young female participants aged 19-20 years, most of whom were 20 years old (76%). The majority had a height of 151-160 cm (44%) and a weight of 41-50 kg (40%). Most participants (76%) had a normal BMI, while 12% were underweight and 12% were overweight. Table 1 presents the sociodemographic and anthropometric characteristics of the participants and the distribution of age, height, weight, and body mass index.

**Table 1:** Sociodemographic characteristics of the participants (n = 25).

Variable	Category	Frequency (f)	Percentage (%)
Age (years)	19	6	24
	20	19	76
Height (cm)	145-150	5	20
	151-160	11	44
	161-165	9	36
Weight (kg)	30-40	8	32
	41-50	10	40
	51-60	7	28
Body mass index (BMI)	<18.5	3	12
	18.5-24.9	19	76
	25-30	3	12

Menstrual and reproductive health profile

**Figure 1:**
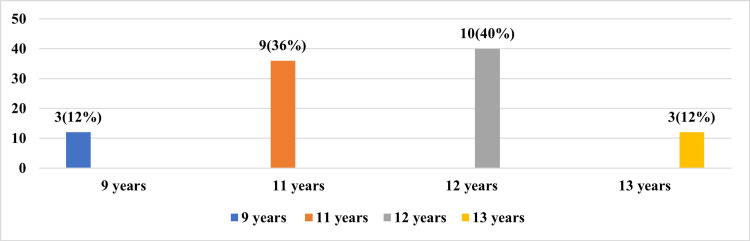
Distribution of age of menarche among the participants (n = 25).

The age at menarche of the participants was between nine and 13 years, with the highest proportion of 40% having a menarche age of 12 years and 36% at the age of 11 years. The presence of regular menstrual cycles was noted by 56% of the respondents, and irregular menstrual cycles were also reported by 44% of respondents, which suggests that the presence of menstrual disturbances is very prominent. Among individuals with irregular menstrual cycles, three (27%) had increased cycle frequency, six (55%) experienced amenorrhea lasting several months, and two (18%) had an increased duration of menstrual bleeding. These results point out the existence of menstrual abnormalities in a significant percentage of young women; this could be a sign of some underlying endocrine malfunction. Figure 1 presents the distribution of the participants based on the age of menarche. Most of the participants attained menarche at the age of 12 years.

**Figure 2:**
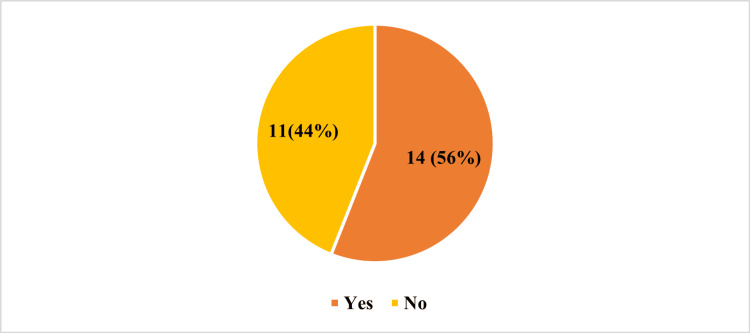
Distribution of regularity of menstrual cycle (n = 25).

Figure 2 depicts the frequency of regular menstrual cycles and irregular menstrual cycles among the participants, with the majority reporting that they had a regular menstrual cycle as opposed to those with irregular menstrual cycles.

Premenstrual symptoms among the participants

**Figure 3:**
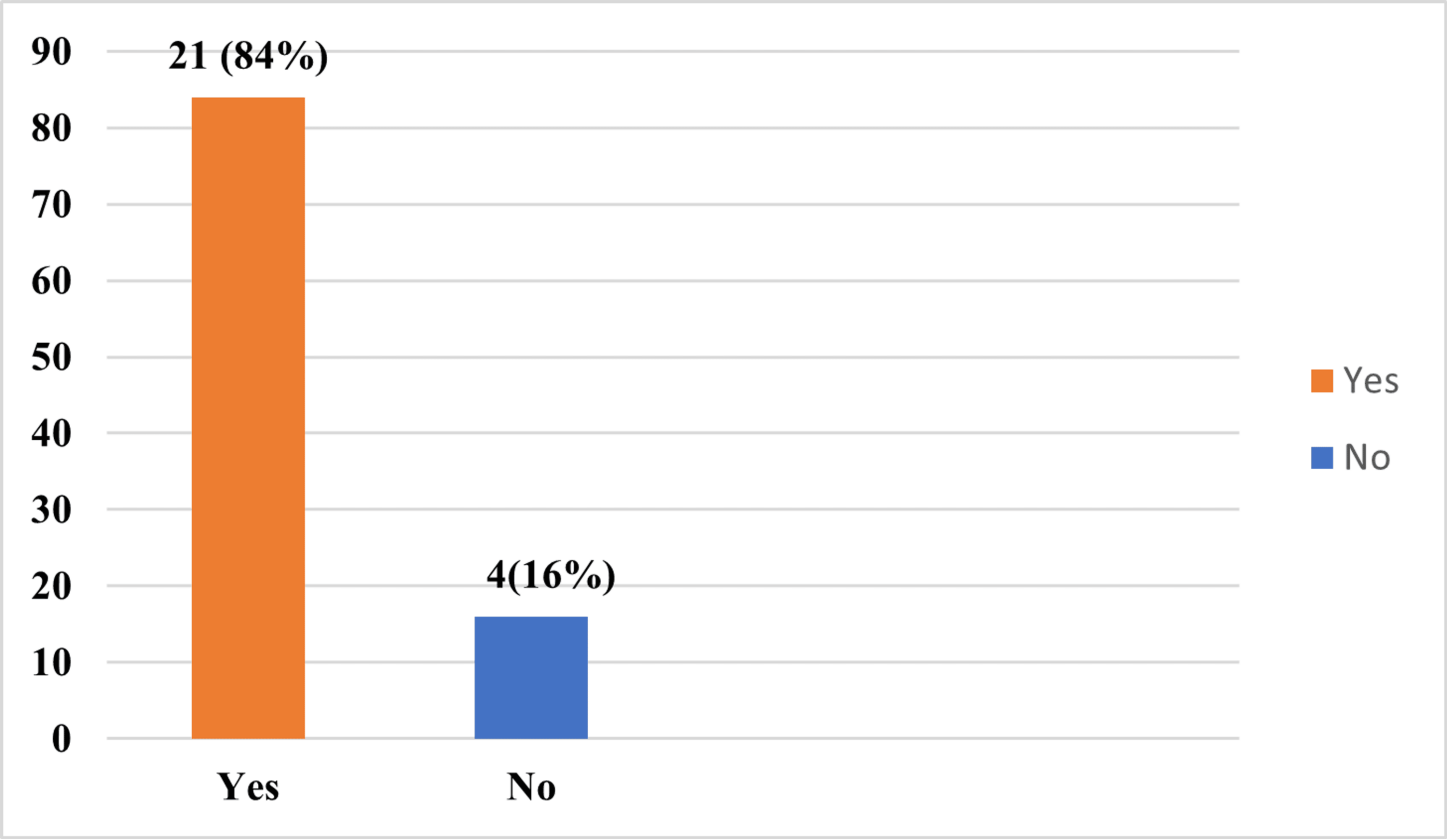
Distribution of premenstrual symptoms among the participants (n = 25).

Most of the respondents (84%) complained of premenstrual symptoms, implying that cyclical symptomatology was predominant in the study population. Abdominal cramps, acne, and low back pain (52.38%) were the symptoms that came next, followed by breast tenderness (42.86%). A smaller percentage of them reported systemic symptoms such as bloating, nausea, and headache (28.57%). Such findings suggest that the pressure of premenstrual symptoms among young females is high and can be associated with hormonal fluctuations. Figure 3 shows the distribution of the participants as per their reporting of premenstrual symptoms. The majority of participants reported premenstrual symptoms.

Family history and comorbid conditions

**Figure 4:**
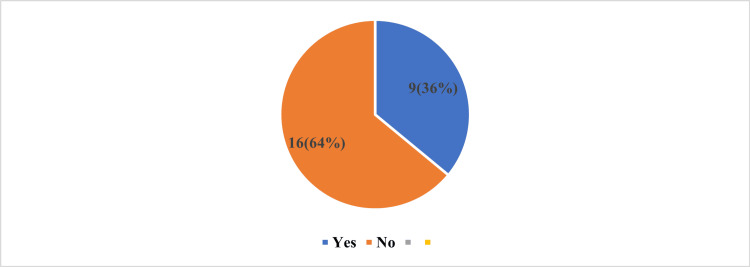
Distribution of family history of menstrual irregularity (n = 25).

A total of 36% of participants reported that they had a family history of menstrual irregularity, which indicates a potential genetic or familial predisposition to disorders of reproductive health. A total of 44% of the participants had comorbid conditions, with polycystic ovarian syndrome being the most common (45.46%), followed by thyroid disorders (36.36%) and asthma (18.18%). The presence of endocrine comorbidities supports the correlation between hirsutism and hormonal imbalances. The results highlight the need for a comprehensive clinical assessment of individuals presenting with features suggestive of hyperandrogenism. Figure 4 demonstrates the distribution of the participants based on their family history of menstrual irregularity, with most of them indicating that they had no such history.

**Figure 5:**
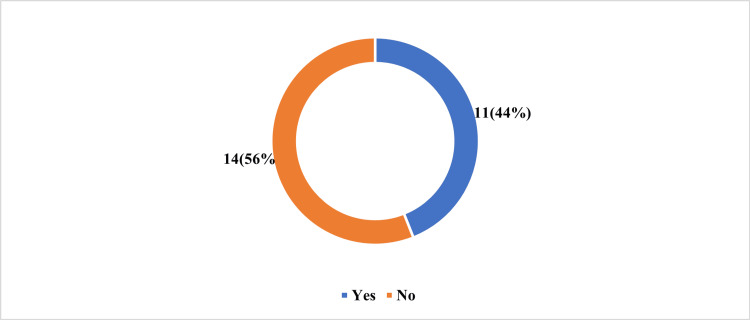
Distribution of comorbid conditions among the participants (n = 25).

Figure 5 presents the distribution of participants based on the presence of comorbid conditions, with the majority of participants not having any comorbidity.

Medication use and cosmetic practices

**Figure 6:**
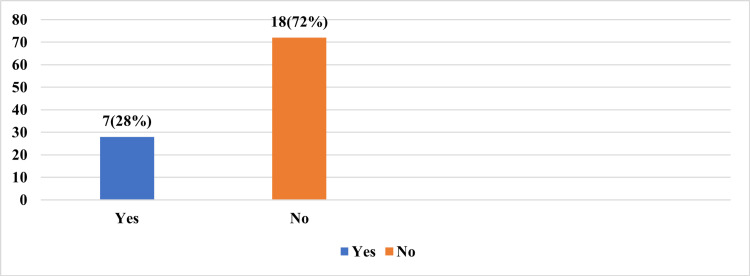
Distribution of regular medication intake (n = 25).

The frequency of taking medication was 28%. Most participants were taking thyroid medication like levothyroxine (57.14%). A lesser percentage were taking metformin for polycystic ovarian syndrome, and some indicated other treatments. Hair removal cosmetic procedures were common, with 56% of participants reporting the use of threading and waxing. Only a small percentage of participants chose laser treatment. The results of these studies show that many of the respondents are actively involved in the treatment of the symptoms of hirsutism or other related conditions, including both medical and cosmetic treatment. Figure 6 shows the percentage distribution of participants based on regular medication intake, with most participants not taking any regular medication.

**Figure 7:**
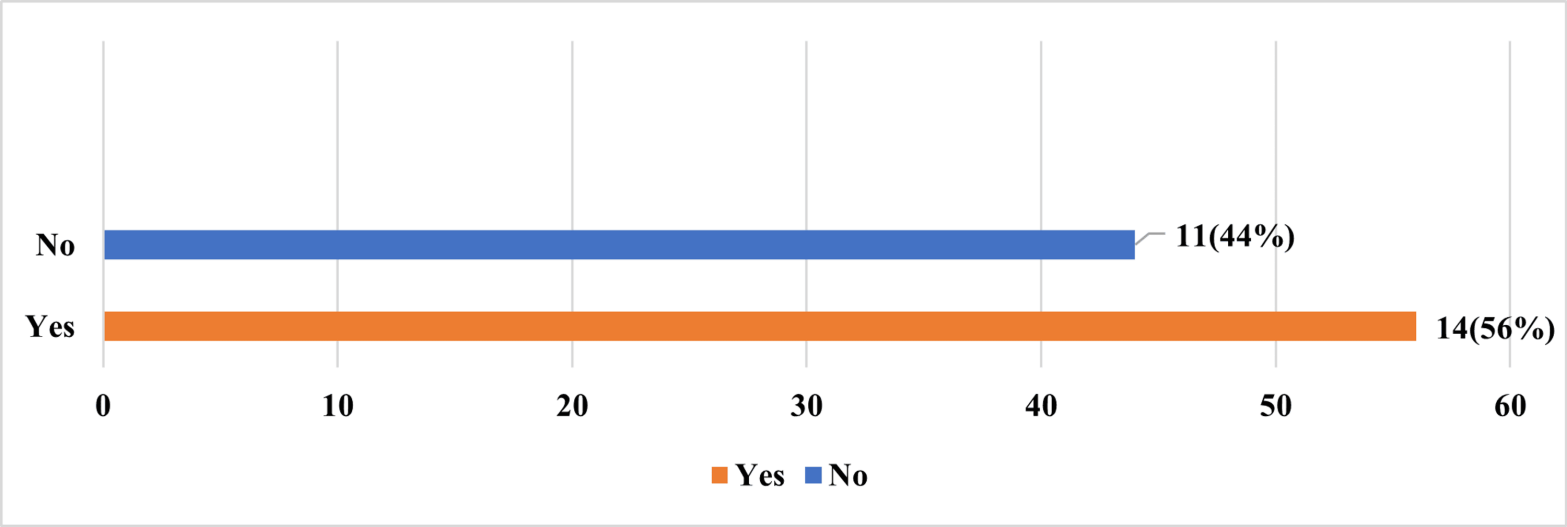
Distribution of cosmetic treatment practices (n = 25).

Figure 7 displays the distribution of participants based on cosmetic treatment practices, with a greater percentage of participants reporting the use of cosmetic treatments.

Distribution of hirsutism severity (mFG score)

**Figure 8:**
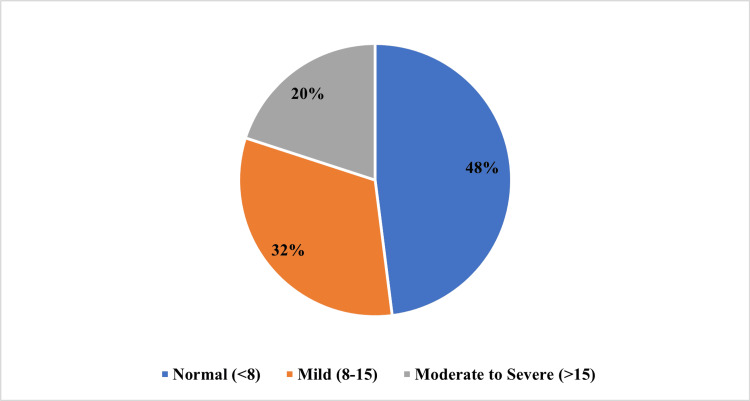
Distribution of hirsutism scores among the participants (n = 25).

The evaluation based on the mFG scoring system [21] revealed that 48% of the sample had normal hair growth (<8), and 32% had mild hirsutism (scores 8-15). This severity grading represents the primary outcome of the study and provides preliminary data on the distribution of normal, mild, and moderate-to-severe hirsutism in this young non-clinical sample. Interestingly, one out of five had moderate to severe hirsutism (>15), which shows that a group of people has clinically significant androgen excess. These outcomes imply that the majority of participants have normal or mild hirsutism severity, although a considerable proportion may require further clinical evaluation. The distribution highlights the importance of mFG scoring as a screening method in the establishment of various levels of hirsutism in young women. Figure 8 shows the percentage distribution of hirsutism severity among the participants, with nearly 50% having normal scores, while the remaining participants had mild or moderate-to-severe scores.

Association of mFG score with selected variables

The association of the mFG score with selected variables was assessed among young females. Spearman’s rank correlation was used for BMI and age of menarche because these were continuous or ordinal variables and the pilot sample size was small. The Mann-Whitney U test was used for categorical variables to compare mFG scores between two independent groups. A p-value of <0.05 was considered statistically significant. Given the pilot design and small subgroup sizes, the statistical findings were interpreted as preliminary and exploratory rather than confirmatory. The analysis showed that BMI, family history of hirsutism, and comorbid conditions were significantly associated with the severity of hirsutism among the participants. BMI demonstrated a strong positive correlation with hirsutism scores (r = 0.754, p = 0.000013), and family history of hirsutism (U = 111.5, p = 0.023) and the presence of comorbid conditions (U = 112.5, p = 0.049) were also found to have statistically significant associations. In contrast, age of menarche, regularity of menstrual cycle, premenstrual symptoms, and regular treatment did not show significant associations with hirsutism severity, as their p-values were greater than 0.05 (Table 2).

**Table 2:** Association of modified Ferriman-Gallwey (mFG) score with selected socio-demographic variables in young females (N = 25). Data are presented as test statistic values with corresponding p-values. rₛ: Spearman’s rank correlation coefficient [22]. U: Mann-Whitney U test statistic [23]. * Significant at the 0.05 level. ** Nonsignificant at the 0.05 level.

S. No.	Demographic variables	Test statistic	p-value
1	BMI	rₛ = 0.754	0.000013*
2	Age of menarche	rₛ = 0.364	0.074**
3	Regularity of the menstrual cycle	U = 82.0	0.8**
4	Premenstrual symptoms	U = 30.0	0.381**
5	Family history of hirsutism	U = 111.5	0.023*
6	Co-morbid condition	U = 112.5	0.049*
7	Regular treatment	U = 64.0	0.975**

## Discussion

The prevalence and severity of hirsutism among young females in the present pilot study were also measured using the mFG scoring system, which is a standardised and well-established clinical technique for measuring androgen-dependent hair growth. The main contribution of this pilot study is the demonstration that structured mFG-based grading can be feasibly applied in a college-based setting to categorise hirsutism severity and identify the proportion of participants falling into normal, mild, and moderate-to-severe categories. These findings suggest that even within a non-clinical group of young adults, varying degrees of hirsutism were observed. This indicates that hirsutism may be present beyond clinically diagnosed populations and highlights the importance of recognising it as a condition that can affect the general young female population. The quantified scoring system allowed hair growth patterns to be measured objectively and consistently, which aligns with recommended approaches for evaluating hyperandrogenic conditions in women of reproductive age [24-26]. The demographic characteristics of the study group revealed a narrow age range, with the majority of participants being 20 years old and having a mean BMI within the normal range. This is a sign that the presence of hirsutism and its surrounding factors may also be exhibited in patients who are not characterised by the presence of metabolic issues. Age of menarche falls within the normal physiological range in this study; however, the proportion of menstrual irregularities and premenstrual symptoms is also large. The clinical implications of such findings include that menstrual dysfunction is usually associated with undiagnosed endocrine illnesses, particularly hyperandrogenic disorders like PCOS [18].

The endocrine pathophysiology of hirsutism is also demonstrated by the fact that nearly half of the respondents were found to have comorbid conditions, particularly thyroid disorders and PCOS. Thyroid dysfunction and PCOS can also be recognised as typical contributors of hormonal imbalance and may result in androgen excess or modify peripheral androgen sensitivity. The comorbidity of these conditions underlines the importance of a complete clinical screening of people with hirsutism, especially at the onset of adulthood, when the introduction of interventions can prevent the effects of the metabolic and reproductive dysfunctions in the long term [27]. The severity of hirsutism distribution in the current study has shown that almost 50% of the respondents had normal scores, but a considerable number had mild to moderate and severe hirsutism. It is also particularly interesting to find that 20% of the study participants had moderate to severe hirsutism, which can be subjected to additional endocrinological tests and specific care. These results suggest that hirsutism cannot be only a cosmetic issue, but it might be an initial clinical sign of hormonal dysregulation [28].

The comparison to the past studies shows similarities and differences. The investigation of women in Kosovo showed a high level of hirsutism with menstrual aberrations and a considerable number of women with positive family history [24]. Menstrual cycle distribution and correlation to reproductive age groups of the said study are in agreement with the present results, and it is presumed that hirsutism is found to be common during late adolescence and early adulthood [24]. However, there can be variation in severity distribution and prevalence rates, which may be a result of differences in the sample size, ethnicity, and study design. The smaller sample size of the present pilot study does not permit comparing it directly, but provides useful preliminary results. This pilot study is relevant because it provides preliminary community-based data on hirsutism severity among young females and demonstrates the feasibility of using mFG scoring in a non-clinical college population. The findings may guide future larger studies by informing sample-size planning, identifying variables for further evaluation, and supporting the inclusion of clinical, biochemical, hormonal, ultrasonographic, and psychosocial assessments in future hirsutism research.

The popularity of cosmetic procedures among the participants is a symptom of the psychosocial and aesthetic weight of hirsutism. Excess hair growth in women often affects body image, self-esteem, and social confidence because it differs from socially accepted feminine appearance standards. Many women experience embarrassment, anxiety, emotional distress, and social withdrawal, leading to frequent use of cosmetic hair removal methods to conceal the condition. Hirsutism may also negatively influence interpersonal relationships, daily activities, and overall quality of life, particularly among young adults who are more sensitive to appearance-related concerns. Therefore, hirsutism should be recognised not only as a cosmetic or endocrine condition but also as an important factor affecting psychological well-being and quality of life [29].

The majority of the participants reported on treatment procedures such as waxing and threading, and this indicates that they are less focused on treating the causes and more on controlling the symptoms. This fact explains why more awareness should be created regarding the medical assessment and evidence-based treatment solutions. Visible or excessive hair growth is associated with significant psychological distress and impaired quality of life. Since medical management of the underlying disorder often requires a prolonged duration to achieve noticeable improvement in clinical features, adjunctive cosmetic therapies frequently play an important role in symptom management and psychosocial well-being [27].

The clinical and general health implications of the findings are that screening of hirsutism and endocrine problems among young females is very important even in the community setting. The mFG scoring mechanism was a good tool to be employed during the first phases of assessment, as it is not intrusive and is cost-effective. However, the method is inherently subjective, as participants may interpret the severity of hair growth differently, leading to variability in scoring. Unlike clinician-administered assessments, self-reported scoring lacks clinical standardisation and may therefore reduce consistency and reproducibility. Cultural and ethnic perceptions regarding body hair may also influence how individuals perceive and rate hair growth severity. In addition, cosmetic practices such as shaving, waxing, threading, or laser hair removal can alter hair visibility and affect scoring accuracy. Feelings of embarrassment, social stigma, or personal concerns about appearance may contribute to underreporting or overreporting of symptoms, while differences in awareness and understanding of the scoring method may further influence the accuracy of self-assessment [20].

Limitations and future recommendations

There are several limitations associated with this study that should be taken into consideration when deciphering the results. The small sample size (n = 25) and pilot study design suppress the extrapolation of the findings to a larger population. Convenience sampling has the risk of selection bias, which decreases representativeness. The subjective variability and misclassification could have been caused by self-assessment of hirsutism by the mFG score.

The absence of hormonal or biochemical assessment of androgen levels represents a major methodological limitation, as it restricts confirmation of hyperandrogenism and limits the ability to establish definitive etiological associations with hirsutism. This limitation is particularly important because clinical mFG scoring alone cannot confirm biochemical hyperandrogenism or determine the exact endocrine pathology underlying hirsutism. Consequently, the findings rely primarily on clinical and self-reported assessments without biochemical validation. In addition, ultrasonographic evaluation of the abdomen and pelvis was not performed; therefore, associated conditions such as PCOS and other underlying reproductive or endocrine abnormalities could not be confirmed. Furthermore, the cross-sectional study design does not permit causal inferences or evaluation of temporal relationships between associated factors and the development of hirsutism. Hence, future longitudinal studies incorporating hormonal, biochemical, and ultrasonographic investigations are required to provide a more comprehensive understanding of the condition and its associated disorders.

Future research ought to employ greater and more heterogeneous groupings through probability sampling to increase generalizability. Biochemical and hormonal profiling should be incorporated to be able to correlate clinical results with endocrine abnormalities. It is advisable to use longitudinal studies that can evaluate the progression and results of treatment. Clinician-based scoring, being more standardised, could enhance the reliability and consistency of assessment. Future studies may further improve accuracy by incorporating validated inter-rater assessment protocols and trained evaluators to minimise observer variability and strengthen the reproducibility of the mFG scoring system. Multicentric studies incorporating clinical, metabolic, and psychosocial parameters would yield a more detailed picture of hirsutism.

## Conclusions

This pilot quantitative cross-sectional study primarily assessed the grading and severity of hirsutism among young females using the mFG scoring system and provided baseline data on its clinical presentation in this population. In this sample, 48% of participants had normal mFG scores, 32% had mild hirsutism, and 20% had moderate-to-severe hirsutism, showing that the study directly addressed the grading of hirsutism severity as stated in the title. The findings demonstrated that a notable proportion of participants exhibited mild to severe grades of hirsutism, highlighting the usefulness of the scoring system in identifying variations in hair growth severity. These findings suggest that hirsutism may have clinical relevance as a possible indicator of underlying hormonal or endocrine imbalance rather than being solely a cosmetic concern. The mFG scoring system was found to be a practical, non-invasive, and cost-effective tool for community-based screening and preliminary grading of hirsutism among young females. The findings of this pilot study primarily support the feasibility of using the scoring system in non-clinical settings for early identification and assessment of hair growth patterns. However, the subjective nature of the tool and variations among individuals and ethnic groups should be considered while interpreting the results. The results suggest that early diagnosis, systematic assessment, and a multidisciplinary approach to management are necessary to minimise the risk of metabolic, reproductive, and psychosocial complications in the long run. Because of the pilot design, small convenience sample, self-reported scoring, restriction to females aged 18-24 years, and absence of biochemical or ultrasonographic confirmation, the findings should be interpreted cautiously. Larger, well-designed studies with representative sampling, clinician-based scoring, hormonal assessment, biochemical testing, ultrasonographic evaluation, and psychosocial assessment are required to validate these preliminary findings and clarify underlying endocrine associations.
